# The Rights of People With Disabilities in Policy Development: Comment on "How Did Governments Address the Needs of People With Disabilities During the COVID-19 Pandemic? An Analysis of 14 Countries’ Policies Based on the UN Convention on the Rights of Persons With Disabilities"

**DOI:** 10.34172/ijhpm.8736

**Published:** 2025-03-03

**Authors:** Olaf Kraus de Camargo

**Affiliations:** ^1^Department of Pediatrics, Faculty of Health Sciences, McMaster University, Hamilton, ON, Canada.; ^2^CanChild Centre for Childhood Disability Research, Hamilton, ON, Canada.

**Keywords:** Universal Design, Inclusion, Text Mining

## Abstract

Shikako et al analysis highlights that needs of persons with disability (PwD) were often overlooked, with policies primarily focused on general population health measures rather than specific accommodations for PwD. This commentary suggests adopting universal design principles in policy development to ensure inclusivity and advocate for maintaining services essential for PwD even during crises. It emphasizes the importance of involving PwD in policy-making processes and enhancing data collection for better policy analysis and concludes with recommendations for creating more inclusive policies, stressing the need for international collaboration and the integration of PwD needs into all policy levels.

## Introduction

 All policies should consider their impact on health and consider that impact for all people in society.^1^ Disability has traditionally been considered an outcome of health interventions (one to be avoided) as is expressed using the term “burden” of disease in the disability-adjusted life years.^2^ Modern understanding of disability informed by the World Health Organization (WHO) framework of the International Classification for Functioning, Disability and Health, defines disability and functioning as extremes on a continuum and being disabled is a personal characteristic such as gender or race, needing to be considered as such in health statistics and policies.^3^ This justifies analyzing policies through a disability lens as Shikako and colleagues did.^4^

 The COVID-19 pandemic highlighted the gaps in risk and emergency planning for persons with disability (PwD). Article 11 of the United Nations Convention on the Rights of Persons with Disabilities (UNCRPD) obligates states to take all necessary measures to ensure the protection and safety of PwD in situations of risk and emergencies.^5^ The analysis by Shikako et al showed that while many countries did implement measures to protect the general population, the specific needs of PwD were often overlooked.^4^ This oversight might have resulted in higher rates of morbidity and mortality among this vulnerable group.

 For instance, in the United States and the United Kingdom, the excess deaths were significantly higher than reported COVID-19 deaths, suggesting that many PwD may have died from causes indirectly related to the pandemic, such as lack of access to essential services and medical care.^6^ Gleason and colleagues’ findings that intellectual disability was a strong independent risk factor for COVID-19 mortality further underscore the urgent need for inclusive policies that protect PwD during health crises.^7^

## Non-essential Services and Social Participation

 The pandemic also exposed the critical role of services deemed “non-essential” in the lives of PwD. While the WHO classified all rehabilitation services as essential, many administrations did not consider that limiting the access to wheelchair repairs, sign language interpreters, and personal assistants created barriers for PwD to live independently and participate fully in society. The disruption of these services during the pandemic had severe consequences for the mental and physical health of PwD.^8-10^

 Prvu Bettger et al emphasized the importance of maintaining essential rehabilitation services across the care continuum to support PwD during the pandemic.^8^ Similarly, Cadwgan et al and Currie et al highlighted the mental health challenges faced by children with neurodevelopmental disabilities and their families, exacerbated by the pandemic’s impact on service availability.^9,10^ Zybarth et al further illustrated how the pandemic affected patients with rare diseases, stressing the need for resilient healthcare systems that can continue to provide essential services even during crises.^11^ These findings make the case for the need of a differentiated definition of what is “essential” for whom.

## Universal Design and Policy Development

 The concept of universal design offers a promising framework for inclusive policy development. Originating in the field of architecture, universal design principles advocate for the creation of environments and services that are accessible to all people, regardless of their abilities or disabilities.^12^ Ostroff’s work on universal design highlights its evolution from physical spaces to broader applications in education, business, and social services.^13^

 In the context of policy development, Bickenbach’s reflection on universally designed social policy suggests that such an approach can reduce the need for specific disability-targeted policies, which are often administratively burdensome and exclusionary.^14^ By integrating universal design principles, policy-makers can create more inclusive policies that address the needs of all citizens, including PwD. An example how this results in inclusive and accessible services for all is the recent Accessibility for Ontarians with Disability Act in Canada (https://www.aoda.ca).

 When do policies get revised? Developing policies usually involves a process of gathering input from a multitude of stakeholders and experts in the field. Once policies have been implemented not always, they have an “expiration date” or an established timeline when they should be revised.^15^ Revision might become necessary in regular intervals due to changes in society, changes of the situation that needs to be addressed, availability of services, new technological developments and changes in other related policies. Policies also might need to be revised when the intended impact on health is not being achieved. Besides such direct assessment of one policy, it might also be informative to compare policies from different nations to learn from them and to develop benchmarks of what seem to be essential features to be addressed. This has been nicely demonstrated by Shikako et al with the use of text mining technology.^4^ In the future such technologies with the added capacity of artificial intelligence might allow for even faster comparison and assessment of policies, allowing an accelerated policy-cycle in rapidly changing situations such as the one during the COVID-19 pandemic. For the time being, this needs to become an ongoing process of analysis to proactively update policies in preparation for future emergencies and crises. The pandemic has shown that preparedness is essential.

## Moving Forward: Recommendations

 To better address the needs of PwD during future pandemics or similar crises, the following recommendations can be made based on the findings of Shikako et al and the literature cited:

1. Incorporate Universal Design Principles: Policies should be developed with universal design principles in mind, ensuring accessibility and inclusivity from the outset. This approach can help avoid the pitfalls of targeted policies and create a more equitable society.
2. Maintain Essential Services: Governments must recognize the critical nature of what constitutes essential services for PwD and ensure their continuity during crises. This includes not only medical and rehabilitation services but also social and support services that enable independent living.
3. Enhance Data Collection and Analysis: Techniques like text mining, as used by Shikako et al, can provide valuable insights into the effectiveness of policies and identify gaps. Such techniques could already be employed during the policy development process to ensure alignment with the UNCRPD. Once implemented, policies should be assessed on their health impact to adapt them accordingly.
4. Engage PwD in Policy Development: PwD should be actively involved in the policy development process. Their firsthand experiences and perspectives are invaluable in creating policies that truly meet their needs. The revenue agency of the government of Canada has established a Disability Advisory Committee which is composed by professionals from various fields, including PwDs (https://www.canada.ca/en/revenue-agency/corporate/about-canada-revenue-agency-cra/disability-advisory-committee.html).
5. Strengthen International Collaboration: The global nature of the pandemic underscores the importance of international collaboration in policy development. Countries can learn from each other’s successes and challenges to create more robust and inclusive policies. Sustainable Development Goal 17 envisions strengthening global partnerships (https://sdgs.un.org/goals). Countries commit to work on national sustainable development strategies and comparative policy analysis focusing on inclusion and participation of all citizens could become part of these goals.

## Conclusion

 The analysis by Shikako et al provides a crucial examination of how governments addressed the needs of PwD during the COVID-19 pandemic.^4^ While significant gaps were identified, the findings also offer a roadmap for creating more inclusive and effective policies in the future. By incorporating universal design principles, defining essential services for PwD, enhancing data collection, engaging PwD in policy development, and strengthening international collaboration, we can better protect and support PwD in times of crisis and beyond.

## Ethical issues

 Not applicable.

## Conflicts of interest

 Author declares that he has no conflicts of interest.

## References

[1] De Leeuw E, Peters D (2015). Nine questions to guide development and implementation of Health in All Policies. Health Promot Int.

[2] Krahn G. Where is disability in global public health? In: Oxford Research Encyclopedia of Global Public Health. Oxford, UK: Oxford University Press; 2021.

[3] Fortune N, Madden RH, Clifton S (2021). Health and access to health services for people with disability in Australia: data and data gaps. Int J Environ Res Public Health.

[4] Shikako K, Lencucha R, Hunt M (2023). How did governments address the needs of people with disabilities during the COVID-19 pandemic? An analysis of 14 countries’ policies based on the UN convention on the rights of persons with disabilities. Int J Health Policy Manag.

[5] UN General Assembly. Convention on the Rights of Persons with Disabilities and Optional Protocol. New York: United Nations; 2007. 10.1515/9783110208856.203 18348362

[6] Islam N, Shkolnikov VM, Acosta RJ (2021). Excess deaths associated with COVID-19 pandemic in 2020: age and sex disaggregated time series analysis in 29 high income countries. BMJ.

[7] Gleason J, Ross W, Fossi A, Blonsky H, Tobias J, Stephens M (2021). The devastating impact of COVID-19 on individuals with intellectual disabilities in the United States. NEJM CatalInnov Care Deliv.

[8] Prvu Bettger J, Thoumi A, Marquevich V (2020). COVID-19: maintaining essential rehabilitation services across the care continuum. BMJ Glob Health.

[9] Cadwgan J, Goodwin J, Arichi T (2022). Care in COVID: A qualitative analysis of the impact of COVID-19 on the health and care of children and young people with severe physical neurodisability and their families. Child Care Health Dev.

[10] Currie G, Finlay B, Seth A (2022). Mental health challenges during COVID-19: perspectives from parents with children with neurodevelopmental disabilities. Int J Qual Stud Health Well-being.

[11] Zybarth D, Brandt M, Mundlos C, Inhestern L (2023). Impact of the COVID-19 pandemic on health care and daily life of patients with rare diseases from the perspective of patient organizations - a qualitative interview study. Orphanet J Rare Dis.

[12] Preiser WF, Smith KH. Universal Design Handbook. New York, NY: McGraw Hill; 2011:492.

[13] Ostroff E. Universal design: an evolving paradigm. In: Preiser WF, Smith KH, eds. Universal Design Handbook. New York, NY: McGraw Hill; 2011.

[14] Bickenbach J (2014). Universally design social policy: when disability disappears?. DisabilRehabil.

[15] Boehmke FJ, Desmarais BA, Eastman A (2023). SPRC19: a database of state policy responses to COVID-19 in the United States. Sci Data.

